# Effects of Dietary Tea Powder on the Growth Performance, Carcass Traits, and Meat Quality of Tibetan Pig × Bama Miniature Pigs

**DOI:** 10.3390/ani11113225

**Published:** 2021-11-11

**Authors:** Zhaoming Yan, Yinzhao Zhong, Yunju Yin, Yehui Duan, Wenlong Wang, Lingyu Zhang, Qiuping Guo, Qinghua Chen, Fengna Li

**Affiliations:** 1 Laboratory of Animal Nutritional Physiology and Metabolic Process, Institute of Subtropical Agriculture Chinese Academy of Sciences, Key Laboratory of Agro-Ecological Processes in Subtropical Region, Hunan Provincial Engineering Research Center for Healthy Livestock and Poultry Production, Scientific Observing and Experimental Station of Animal Nutrition and Feed Science in South-Central, Ministry of Agriculture, Changsha 410125, China; yanzmmail@163.com (Z.Y.); yinyj1124@163.com (Y.Y.); duanyehui@isa.ac.cn (Y.D.); wangwenlong2012@yeah.net (W.W.); zly_012@163.com (L.Z.); gqp9210@163.com (Q.G.); 2 College of Animal Science and Technology, Hunan Agricultural University, Changsha 410128, China; 3 Shanghai Key Laboratory of Regulatory Biology, Institute of Biomedical Sciences and School of Life Sciences, East China Normal University, Shanghai 200241, China; yinzhaoz@163.com; 4 Laboratory of Animal Nutrition and Human Health, School of Biology, Hunan Normal University, Changsha 410018, China; 5 University of Chinese Academy of Sciences, Beijing 100049, China

**Keywords:** tea powder, growth performance, carcass traits, meat quality, native pig

## Abstract

**Simple Summary:**

In China, and even the world, the animal husbandry industry is facing the problem of a shortage of feed ingredients, and even humans and animals compete for food. The development of unconventional feed resources is an urgent task. In our previous work, we analyzed the nutritional value of tea powder and believed that it has development potential. This study investigated the effects of supplementing different levels of tea powder in diets on pig growth performance, meat quality, and antioxidant properties. These results provide a preliminary basis for the application of tea powder as a supplement in animal husbandry.

**Abstract:**

This study was conducted to evaluate the effects of tea powder in diets on the growth performance, meat quality, muscular amino acid, fatty acid profile, and serum biochemical indices of pigs. A total of 120 local Chinese pigs (Tibetan × Bama miniature pigs) were randomly assigned to five treatment groups, each with six pens and four pigs per pen. During a 60-day experiment, these groups of pigs were fed the normal diet and the diet supplemented with 1%, 2%, 4%, and 6% tea powder, respectively. The results showed that the supplementation of tea powder did not affect the growth performance of pigs. However, the dietary tea powder inclusion decreased (*p* < 0.05) the average fat thickness, total fat, and abdominal fat, and increased (*p* < 0.05) the total muscle as well. In addition, the dietary 2% tea powder decreased (*p* < 0.05) the muscle lightness (L*) and yellowness (b*). Compared with the control group, the dietary supplementation with 1%, 2%, and 4% tea powder raised (*p* < 0.05) the total amino acids (TAA) and essential amino acids (EAA), and dietary 4% and 6% tea powder increased (*p* < 0.05) the C20:5n3 in the muscle tissue. Furthermore, the serum lipid metabolism-related biochemical indices and mRNA expression levels were improved with the addition of tea powder. These results indicated that dietary tea powder might improve the carcass traits and meat quality of the Chinese native finishing pigs, but it does not affect their growth performance. Tea powder could be fully developed and reasonably applied as a dietary supplement.

## 1. Introduction

With the continuous improvement of people’s quality of life, the consumer demand has changed. Therefore, the market needs to provide higher quality meat to meet the consumer demand. At the same time, improving the quality of meat products through nutritional adjustments or changing feed ingredients is one of the hotspots in animal nutrition research [1].

Tea has widely spread and rapidly become one of the most popular plant-based beverages around the world because of its rich nutrition and unique taste. Tea contains a variety of nutrients, including tea polyphenols, tea polysaccharides, tea saponin, a wealth of vitamins, and minerals [2]. However, in the process of tea production and processing, more than 20% of waste tea (tea stalks and broken leaves with no commercial value) will be produced, which is not for human consumption. In 2020, China produced more than 2.8 million tons of tea, resulting in more than 500,000 tons of waste tea. If tea that cannot be sold is used as fuel, it will not only waste resources but also pollute the ecological environment. Using tea as a dietary supplement is complementary to the development of animal husbandry and has a protective effect on the natural environment. China’s local pig breeds are rich in resources, each with its own characteristics. In summary, Chinese local pigs generally show several strengths, such as strong fecundity, environmental adaptability, stress resistance, and excellent meat quality. At the same time, the relative low growth rate and lean percentage are two main hinderances in the development of Chinese pigs. Previous studies have shown that the dietary addition of tea powder has successfully enhanced the meat quality of pork and inhibited the effect on oxidation. Tea polyphenols and tea by-products can improve the pig carcass traits while increasing the crude protein content in the muscle [3,4].

The Bama miniature pig is a fat-meat breed of swine in south China, with excellent reproductive performance and tender meat. The Tibetan pig is a lean-meat pig from southwest China, with strong adaptability and body-resistance. Unfortunately, the published articles did not address the appropriate range of tea powder supplements in the feed of Chinese local pigs (Tibetan pig × Bama miniature pig). In addition, the effects of tea powder on the pork quality and carcass fat rate are rarely reported. Therefore, we studied the effects of adding different levels of tea in the feed on the carcass traits, meat quality, and blood biochemical indices of the pigs to find a more efficient diet for this specific population in this specific environment.

## 2. Materials and Methods

All experiment procedures in this study were approved by the Hunan Agricultural University and the Animal Care Committee of the Institute of Subtropical Agriculture, the Chinese Academy of Sciences.

### 2.1. Preparation of Tea Powder and Its Nutritional Components

Tea was purchased from Datong-mountain national forest farm (Changde, Hunan, China). The leaves and stems that cannot be sold were first picked from the tea leaves, cleaned and dried under natural conditions, and then simply stir-fried until the surface of the tea leaves was black, dry, and brittle. The treated tea leaves were placed in a 65 °C constant temperature drying room for 3 days. Then, the leaves were pulverized by a grinder and placed in a dry and dark warehouse to protect the quality of the tea powder from deterioration.

Dry matter content determination: gb6435-86 was used to determine the water content and then subtract from the total composition; determination of crude fat content: SOXHLET extraction method (GB/T6433-1994); determination of crude protein content: Kjeldahl nitrogen determination method (GB/T6432-1994); determination of crude fiber content: acid and alkali washing method (GB/T6434-1994); determination of crude ash content: GB/T6438-1992; determination of gross energy: the use of complete oxidation combustion method in the oxygen bomb type calorimeter; determination of calcium content: EDTA collateral-titration method (GB/T6434-1994); determination of phosphorus content: molybdenum yellow colorimeter method (GB/T6437-1994); nitrogen free extract content determination: calculated by the following formula, nitrogen free extract % = [dry matter % − (crude protein % + crude fat % + crude fiber % + crude ash %)]. According to the above method, the nutritional composition of tea powder was analyzed, and the results are shown in Table 1.

### 2.2. Animals and Experimental Treatments

A total of 120 Tibetan pig × Bama miniature pig crossbreed pigs (both are local Chinese pig breeds, castrated male: female ratio = 1:1) with a mean initial body weight (BW) of 23.61 ± 2.79 kg were used. They were fed with a corn–soybean meal; the basal diet (Table 2) was formulated according to the Chinese National Feeding Standard of Swine (Ministry of Agriculture of the People’s Republic of China, 2004). After 3 days adaption period, these pigs were assigned into 5 treatment groups randomly, each with 6 replicates, and each replicate with 4 pigs. The trial period lasted for a total of 60 days. The control group was provided with basal diet; 4 experimental groups were added: 1%, 2%, 4%, and 6% proportions of tea to the basal diet, respectively.

Every 4 pigs were housed together in 2.5 × 2.5 m cages on the ground. The space is enough for 4 pigs to roam free, simultaneously feed, and intake water. The pigs were fed three times a day, at 8:00 a.m., 1:00 p.m., and 6:00 p.m., respectively. Ear tags were used to distinguish between groups. The initial weight, final weight, and daily feed intake of each pig were recorded; average daily gain (ADG) and average daily feed intake (ADFI) of each pig were measured. The feed conversion then could be obtained from dividing feed intake by animals’ weight (F: G).

### 2.3. Sample Collection

At the end of the test, the remaining feed in the trough was cleaned. Ten pigs were selected randomly for slaughter in different treatment groups and had been taken off feed for 24 h before slaughter. All the samples were transported from the farm to the slaughterhouse (10 km, 30 min) and were sacrificed by using electrical stunning (120 V, 180 Hz, about 3–5 s) followed by exsanguination and dehairing. Then, the butcher removed the heads, tails, hoofs, and internal organs. After these operations, weighed out the carcass weights and calculated the slaughter rate (carcass weight/live body weight); each carcass was divided into two halves.

Blood was collected from the jugular vein of the pigs with a volume of 20 mL before slaughter. After placing the blood samples at r.t. for 30 min, centrifuged them at 3500 r/min for 10 min to separate the serum. The serum was put into 1.5 mL EP tubes and stored at −80 °C for subsequent index detection.

### 2.4. Assessment of Carcass Traits and Meat Quality

The *longissimus dorsi* (LD) muscle (300g/pig) and subcutaneous fat (150g/pig) between the sixth rib and the seventh rib were collected in the right side of the carcass and stored at −20 °C. The left half of the carcass was used to measure the thickness of the backfat between the sixth rib and the seventh rib and also determine the skin thickness and the length of the carcass. After that, the carcass was dissected into muscle, fat, and bones. These parts were then weighed and the results recorded to calculate the total muscle percentage, total fat percentage, and total bone percentage.

Meat quality assessment includes muscle color, 45 min pH, 24 h pH, water-holding capacity, shear force, and cooking loss. Muscle pH was measured at 45 min and 24 h after slaughter by using a hand-held pH meter (pH-Star, Matthäus, Pöttmes, Germany), inserting the pH meter electrode into the muscle parallel to the muscle fibers, and recording the data. An objective color analysis of the LD muscle was performed using a chroma meter (CR410, Konica Minolta Sensing Inc., Tokyo, Japan) with D65 light source, 50-mm aperture, and Φ50 mm measurement area after slaughter (45 min), and the L*(lightness), a*(redness), and b*(yellowness) were performed in two repetitions. The LD muscle removed fat and other tissues, heated it to a central temperature of 70 °C in the water bath, and measured the cutting force of the meat using a shearer (GR-150, The G-R Electric Manufacturing Company LLC., Manhattan, NY, USA) for eight repetitions after cooling. LD muscle was cut into a 1 cm thick piece, heated it in a water bath, and calculated the proportion of cooked meat weight to sample weight after cooling [5]. The remaining section was used to access water-holding capacity. The muscle samples were wrapped in gauze, 18 layers of filter paper were placed on the top and bottom of the sample, and under a constant pressure of 300 N for 5 min (Tenovo Meat-1, Tenovo International Co., Limited, Beijing, China), and the water-holding capacity was expressed as the percentage of the meat weight at the end of squeezing. Some muscle samples were freeze-dried and pulverized; Soxhlet leaching (SOX416 Macro, C. Gerhardt Gmbh&Co., Königswinter, Germany) was taken to calculate the intramuscular fat (IMF) content by the freeze-dried muscle sample weight change. In addition, the crude protein (CP) contents of the muscle sample were measured by using Kjeldahl method (QDN-II, Jiangsu tongjun instrument technology Co. Ltd., Changzhou, China). The detection of IMF and CP refers to the Association of Official Analytical Chemists’ methods [6].

### 2.5. Chemical Analysis of Longissimus Dorsi Tissue

The samples were placed in a vacuum freeze dryer and converted into powder. After pretreatment, the composition and content of free amino acids in muscle samples were detected by using a fully automatic amino acid analyzer (L8800, Hitachi, Tokyo, Japan). In detail, approximately 0.5 g of the freeze-dried muscle samples were weighed, homogenized with 2–5 mL of 0.01 N hydrochloric acid, and subjected to ultrasonic extraction. The supernatant was transferred and an equal volume of n-hexane was added to remove fat. After filtration, an 8% sulfosalicylic acid solution was added and left to stand overnight, and 1ml sample was centrifuged and passed through a 0.45 µm membrane, then detected by a fully automatic amino acid analyzer. The content and composition of fatty acids in muscle were measured by gas chromatography (6890N, Agilent Technologies Co. Ltd., Santa Clara, CA, USA).

Approximately 0.5 g of the freeze-dried muscle samples were extracted in a solution of chloroform and methanol (1:1, *v*/*v*); the fatty acid methyl esters were prepared using KOH/methanol. The analysis of fatty acid methyl esters was determined with an Agilent 6890N gas chromatographer equipped with SP2560 column (100 m × 250 μm × 0.2 μm). By comparing with the standard (sigma chemical), the retention time of each fatty acid peak corresponds to it. The concentration of each fatty acid was quantified based on the peak area and calculated as a percentage of the total fatty acids.

### 2.6. Serum Biochemical Indices

Serum enzyme activities of the alanine transaminase (ALT), aspartate transaminase (AST), alkaline phosphatase (ALP), lipase (LPS), serum concentrations of creatinine (CREA), blood glucose (GLU), triglyceride (TG), total cholesterol (CHOL), high-density lipoprotein (HDL), and low-density lipoprotein (LDL) were analyzed using a CX Automatic Biochemical Analyzer (The Beckman Company, Brea, CA, USA) and commercial kits (Leadman Biochemistry Technology Company, Beijing, China) according to the company instruction books.

### 2.7. Serum Antioxidative Parameters

Serum activities of the total antioxidant capacity (TAO-C), superoxide diamutase (SOD), glutathione peroxidase (GSH-Px), and malondiadehyde (MDA) were analyzed using colorimetric methods with a spectrophotometer (Biomate 5, Thermo Electron Corporation, Rochester, NY, USA). The commercial kits were purchased from Nanjing Jiancheng Bioengineering Institute (Nanjing, Jiangsu, China) and used with reference to the instructions.

### 2.8. Quantitative Real-Time PCR Analysis

The quantitative real-time PCR was conducted as described in our previous study [7]. The RNA was isolated from the LD muscle tissue using TRIzol reagent (Invitrogen, Carlsbad, CA, USA), and the test operation was carried out in accordance with the instructions. The ABI7900HT real-time PCR system (Applied Biosystems, Branchburg, NJ, USA) was used for quantitative real-time PCR to determine the relative mRNA expression level of selected genes.

Primers of the target genes were designed using Primer 5.0 software (Table 3). In order to regulate the mRNA expression level of target genes, the housekeeping gene glyceraldehyde-3-phosphate dehydrogenase (GAPDH) was selected as an internal control. The procedure of quantitative real-time PCR is as follows: incubated at 95 °C for 10 min, denatured at 95 °C for 15 s (there are 40 cycles), annealed at 60 °C for 60 s, and extended at 72 °C for 30 s. Using the comparison 2^−ΔΔCt^ method, the threshold cycle (Ct) value of real-time PCR and the value related to the internal control can be used to obtain the mRNA expression level of any unit of the target gene [8].

### 2.9. Statistical Analysis

The data obtained in all experiments were first summarized by Excel software (Microsoft Co., Redmond, WA, USA), and then analyzed by one-way ANOVA using SPSS 20 software (International Business Machines Co., Armonk, NY, USA). The homogeneity test of variance was performed using the Duncan multiple comparisons test in the case of heterogeneity. Orthogonal polynomial contrasts were performed to determine linear and quadratic effects of increasing dietary tea powder on the measured traits. The results were expressed as arithmetic means and SEM; differences were considered statistically significant at *p*-value < 0.05.

## 3. Results

### 3.1. Growth Performance

Table 4 shows that the final weight, ADG, and F:G ratio did not show significant differences between the control and treatment groups. The 2% tea group showed an increase (*p* < 0.05) in the ADFI compared with the 6% tea group, but no significant difference was observed between the control and treatment groups.

### 3.2. Carcass Traits

As shown in Table 5, compared with the control group, both the 2% and 4% tea groups were decreased (*p* < 0.05) in regard to the abdominal fat weight, and the diet supplement with 1% and 2% tea powder demonstrated significantly increased (*p* < 0.05) carcass lengths; the 4% tea group showed a reduction in (*p* < 0.05) the average back fat thickness; the 4% tea group showed a 2.49% increase (*p* < 0.05) in the total muscle percentage; the 2%, 4%, and 6% tea groups showed 4.14%, 5.73%, and 3.55% decreases (*p* < 0.05) in the total fat, respectively. There was no difference (*p* > 0.05) obtained between the control group and the treatment groups in terms of the dressing percentage, skin thickness, and total bone percentage. Furthermore, increasing the level of the dietary tea powder caused the abdominal fat of the pigs to decrease quadratically (*p* < 0.05).

### 3.3. Meat Quality

The effects of the dietary tea powder inclusion level on the meat quality of the pigs are presented in Table 6. The 45 min pH was significantly decreased in the 2% tea group (ANOVA and Quadratic, *p* < 0.05), and the meat lightness (L*) in the 2% and 4% tea groups was decreased (ANOVA and Quadratic, *p* < 0.05) more than in the control group. However, no significant differences were observed in the 24 h pH, meat redness (a*), water-holding capacity, cooking loss, shear force, and IMF, and there is a trend toward significance in the CP (*p* = 0.07).

### 3.4. Amino Acid Profile of LD Muscle

As shown in Table 7, the dietary supplementation with 1%, 2%, and 4% tea powder increased the TAA and EAA in the LD muscle. However, all the treatment groups did not affect the content of the FAA compared with the basal diet group. An analysis of the different kinds of AA revealed that, with the addition of tea powder, the AA content showed a gradual increase, and, when the tea powder was added to 6%, the content decreased. The value of the EAA/TAA in each treatment group was around 40%, and the highest value of the 1% tea powder group was 40.36%. Interestingly, the addition of different levels of tea significantly reduced (ANOVA, Linear and Quadratic, *p* < 0.05) the Asp contents. Furthermore, increasing the level of the dietary tea powder caused the TAA and EAA of the pigs to increase quadratically (*p* < 0.05).

### 3.5. Fatty Acid Profile in LD Muscle Tissue

The fatty acid profile in the LD muscle tissue is shown in Table 8. The results showed no significant difference in the total of SFA, MUFA, and PUFA between the different treatment groups. However, the dietary supplement with 4% or 6% tea powder significantly raised the C20:5n3 in the LD muscle (ANOVA and Linear, *p* < 0.05).

### 3.6. Serum Biochemical Indices

As shown in Table 9, the dietary supplement of 2% tea powder resulted in the higher (*p* < 0.05) enzyme activities of AST and ALP in the serum when compared with the control group, and 6% tea powder significantly reduced the content of CREA in the serum (*p* < 0.05). For the two indices of HDL and LDL, as the amount of dietary tea powder increased, the HDL content gradually increased (*p* < 0.05), but the LDL content showed the completely opposite trend (*p* < 0.05).

### 3.7. Serum Antioxidative Parameters

The effects of the dietary supplement with tea powder on the concentrations of the TAO-C, SOD, GSH-Px, and MDA in the serum of the pigs are presented in Table 10. Only the activity of serum GSH-Px significantly increased in the 2% and 6% tea powder groups compared with the 1% group (*p* < 0.05); however, there was no significant difference between the control group and the treatment groups.

### 3.8. The Relative mRNA Expression Levels of Lipid Metabolism

**Figure 1:**
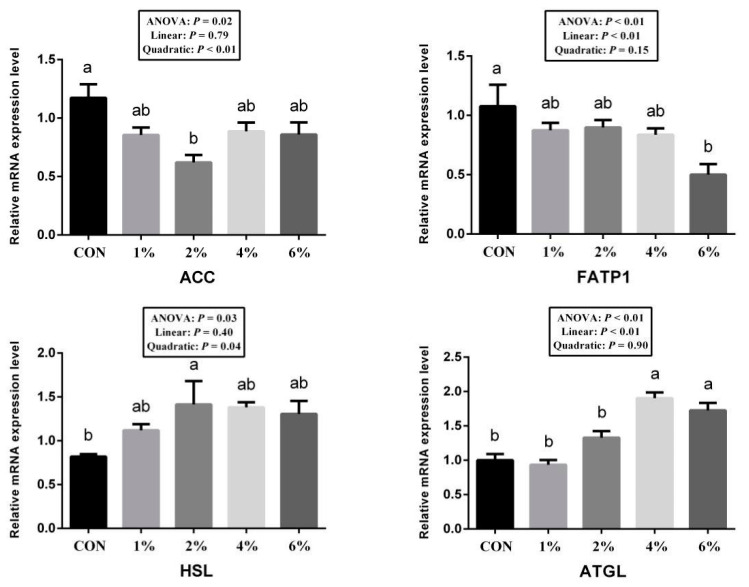
Diet supplement tea powder inhibited fat synthesis and promoted fat catabolism in longissimus dorsi muscle. The relative mRNA expression levels of the key genes related to lipid metabolism include acetyl-CoA carboxylase α (ACC), fatty acid transport protein 1 (FATP1), hormone-sensitive lipase (HSL), adipose triglyceride lipase (ATGL) (*n* = 6). Duncan, linear and quadratic effects for tea powder inclusion levels. Data are expressed as means ± SEM; ab different superscripts indicate significant differences (*p* < 0.05).

As shown in Figure 1, the ACC gene is related to the fatty acid synthesis in the LD muscle, and the use of tea powder significantly decreased the expression of the ACC genes (*p* < 0.05). ATGL and HSL are two important lipases in animals, which break down fats into glycerol and fatty acids. In this study, adding tea powder to the diet can significantly increase the mRNA expression of ATGL and HSL (*p* < 0.05). FATP1 is related to the intake of fatty acids. The addition of 6% tea can significantly reduce the mRNA expression of FATP1 (*p* < 0.05) in the LD muscle.

## 4. Discussion

The polyphenolic compounds belong to the category of biologically active ingredients contained in green tea, which can be divided into flavonoids, flavanols, phenolic acids, and so on. Their biological activities focus on the antioxidant capacity [9], antibacterial capacity [10], lipid metabolism regulation [11], anti-cardiovascular activity [12], anti-cancer activity [13], and gut health-promoting activity [14].

As is commonly known, the quality of meat products determines the consumption habits of consumers. Adding different levels of tea powder to pig diets has varying degrees of impact on pig growth performance, carcass traits, and meat quality. In this study, the dietary tea supplements did not affect the growth performance of the pigs, consistent with previous studies [3]. However, with the increasing amount of dietary tea powder, the ADFI showed a trend to rise before plateauing. The reason might be that tea contains anti-nutritional elements (condensation tannic acid), and tannin acid with a bitter taste can be combined with proteins, sugars, and metal ions to form complexes that are difficult to digest and absorb [15,16,17].

Chinese local pigs have good meat quality and are tolerant to rough feeding but have a higher fat ratio. In previous studies, tea polyphenols can affect the fat deposition by regulating lipid metabolism [18,19] and promoting the expression of PPAR-α, CPTla, and LDLγ [20]. Accordingly, in the present study, the dietary tea supplements reduced the total fat, abdominal fat weight, and average fat thickness. The 4% tea powder group had the most significant effect on the above indicators, with the carcass total fat being reduced by 17.91%, and the total muscle content was increased by 6.20% compared to the control group. The reason for this result may be that 4% of the tea powder has the most obvious regulatory effect on certain lipid metabolism genes, or it promotes the metabolism of TG and glycogen. The same results were obtained after testing the relevant indicators.

The meat quality not only affects consumers’ purchasing decisions but also the meat taste and storage time. Among them, the meat color is the key factor to judge the appearance and freshness of pork [21,22]. Our results suggest that the meat color could be improved by supplementing dietary tea. Previous studies have shown that meat is of the best quality when the content of the IMF in the muscle is about 3% [23,24]. In this study, although the IMF content in the LD muscle of each treatment group reached about 3.5%, the meat quality of the local Chinese pigs was still good. In other words, the addition of tea powder during the whole experiment would not reduce the IMF content.

Meat is an important source of protein with high biological value, providing a variety of amino acid nutrition for human growth and development [25]. The types and composition of amino acids in dietary proteins have an important impact on protein utilization and weight gain [26]. Tea is rich in amino acids, including the theanine found only in tea, which is also the main source of green tea sweetness [27]. The composition and content of amino acids can reflect the protein content of meat products. In the meat quality evaluation system, the flavor of amino acids plays a key role in the flavor of meat [28]. Among them, Asp, Ser, Glu, Gly, and Ala present umami taste [29]. In this study, compared with the control group, each treatment group increased the TAA, EAA, and FAA in the LD muscle, especially for the 4% tea powder group. In addition, in previous studies of the nutritional value of protein and meat, researchers found that the essential amino acid content/total amino acid content (EAA/TAA) of muscles with good meat quality is about 40% [30]. The EAA/TAA of the different treatment groups in this experiment was about 40%, and higher than the control group; the 1% tea group was the highest (EAA/TAA = 40.36%). The polyunsaturated fatty acids are good for human health. Among them, C18:3n3, C20:5n3, and C22:6n3 can reduce cholesterol accumulation in the body [31,32]. The results of this study indicated that, when the dietary tea powder was supplemented at 4% or more, the content of the C20:5n3 showed a significant improvement compared with the control group, improving the nutritional value of the meat.

As an important component, blood is involved in the body composition of people and animals. It is a special connective tissue that carries oxygen and nutrients, discharges metabolic waste, regulates body temperature, and stabilizes vascular pressure. Through the analysis of serum biochemical indices, the degree of digestion and absorption of nutrients and animal health can be judged. In this study, the feeding diets containing more than 2% tea caused an increase in the AST activity, which may be related to the anti-nutritional effects of tannins in tea [33]. Furthermore, when the AST/ALT is greater than one, it can be diagnosed as extensive liver damage [34,35]. The results of this study showed that the AST/ALT were both less than one, and there were no qualitative lesions in the pig liver. Meanwhile, it also suggested that the amount of the dietary tea powder should not be too high. In this study, the ALP activity of the 2% tea powder addition group was significantly higher than that of the control group. The ALP activity of the pigs was significantly positively correlated with the ADG. By improving the activity of the ALP enzyme, it helps to increase the feed intake of the pigs [36]. T-AOC, SOD, and GSH-Px are important indicators that reflect the body’s antioxidant level, also known as the body’s antioxidant enzyme system [37]. Among them, GSH-Px catalyzes peroxides to produce alcohol or water through a reduction reaction. In this process, glutathione is used as a reducing agent to remove oxidizing free radicals. We found that feeding tea powder could increase the activity of GSH-Px in the serum, thereby increasing the antioxidant levels in pigs.

As mentioned above, supplementing the diet with tea powder can help reduce the fat rate and the thickness of subcutaneous fat in pigs. Whether the results are related to the expression level of lipid metabolic genes remains to be studied. ACC is a rate-limiting enzyme that synthesizes fatty acids [38]. According to the results, a dietary supplement of 2% tea significantly reduced the mRNA expression level of the ACC. ATGL and HSL are two important lipases in the animal body. ATGL first hydrolyzes triglycerides into diacylglycerol and one molecule of fatty acid, then *HSL* continues to catalyze the conversion of diacylglycerol into monoacylglycerol and one molecule of fatty acid [39]. In the present study, the tea powder significantly up-regulated the mRNA expression level of the *ATGL* and *HSL*, indicating that tea powder might accelerate the metabolism of fatty acids. Lipid uptake is mainly regulated by fatty acid transporters, such as FATP1, whose overexpression promotes the uptake of fatty acids in cells [40]. In this study, the tea powder significantly reduced the mRNA expression of the *FATP1*, suggesting that it helped reduce the body’s intake of fatty acids.

## 5. Conclusions

In summary, dietary tea powder supplements can improve the carcass traits and meat quality without affecting the growth performance of Tibetan × Bama miniature pigs. It is worth noting that dietary tea powder can be used as a supplement, and this study provides the support of data and a theoretical basis for the effective application of tea powder in the pig industry.

## Figures and Tables

**Table 1:** The nutritional content of tea powder (%).

Item ^1^	Content
Dry Matter	89.23
Gross Energy (MJ/kg)	14.01
CP	23.5
EE	3.91
CF	10.74
NFE	45.41
Ca	0.79
P	0.58

^1^ CP = crude protein; EE = crude fat; CF = crude fiber; NFE = nitrogen free extract; Ca = calcium; P = phosphorus.

**Table 2:** Experimental diets composition and nutrient levels (air-dried basis, %).

Item	Tea Powder Inclusion Level				
	CON ^3^	1%	2%	4%	6%
Ingredient, %					
Corn	61.00	61.20	61.50	60.45	59.30
Soybean meal	22.00	21.80	21.50	20.60	19.70
Wheat bran	14.00	13.00	12.00	12.00	12.05
Tea powder	-	1.00	2.00	4.00	6.00
Lysine	0.10	0.10	0.10	0.10	0.10
CaHPO4	0.70	0.70	0.70	0.70	0.70
Calcium carbonate	0.90	0.90	0.90	0.85	0.85
Salt	0.30	0.30	0.30	0.30	0.30
Premix ^1^	1.00	1.00	1.00	1.00	1.00
Chemical composition ^2^, %					
Digestable energy, MJ/kg	13.60	13.60	13.60	13.52	13.42
Crude protein, %	16.06	16.08	16.06	16.07	16.08
Lysine	0.78	0.78	0.77	0.76	0.74
Methionine + Cystine	0.53	0.52	0.51	0.50	0.49
Threonine	0.51	0.50	0.49	0.48	0.46
Calcium	0.61	0.61	0.62	0.61	0.63
Total phosphorus	0.55	0.54	0.54	0.54	0.54

^1^ Premix, provided per kg of the complete diet: vitamin A, 10,800 IU; vitamin D3, 4000 IU; vitamin E, 40 IU; vitamin K3, 4 mg; vitamin B1, 6 mg; vitamin B2, 12 mg; vitamin B6, 6 mg; B12, 0.05 mg; biotin, 0.2 mg; folic acid, 2 mg; niacin, 50 mg; D-pantothenic acid, 25 mg; copper, 150 mg; iron, 100 mg; manganese, 40 mg; zinc, 100 mg; iodine, 0.5 mg; selenium, 0.3 mg. ^2^ The contents of total energy, crude protein, calcium, total phosphorus, and amino acid composition were analyzed. ^3^ CON = control group (basic fodder), and the following table has the same representation.

**Table 3:** Primers used for quantitative real-time PCR.

Genes	Primers	Sequences (5′ to 3′)	Product Size, bp
ACC	Forward	GGCCATCAAGGACTTCAACC	120
	Reverse	ACGATGTAAGCGCCGAACTT	
ATGL	Forward	ATGTTCCCCAAAGAGACGAC	583
	Reverse	GGCGAAGCGGGTTATGAT	
HSL	Forward	CACAAGGGCTGCTTCTACGG	167
	Reverse	AAGCGGCCACTGGTGAAGAG	
FATP1	Forward	GGAGTAGAGGGCAAAGCAGG	208
	Reverse	AGGTCTGGCGTGGGTCAAAG	
GAPDH	Forward	AAGGAGTAAGAGCCCCTGGA	140
	Reverse	TCTGGGATGGAAACTGGAA	

ACC = acetyl-CoA carboxylase α, ATGL = adipose triglyceride lipase, HSL = hormone-sensitive lipase, FATP1 = fatty acid transport protein 1, GAPDH = Glyceraldehyde-3-phosphate dehydrogenase.

**Table 4:** Effects of dietary supplements with tea powder on growth performance of the pigs.

Item ^1^	Tea Powder Inclusion Level					SEM	*p*-Value		
	CON	1%	2%	4%	6%		ANOVA	Linear	Quadratic
Initial weight, kg	23.49	23.64	23.56	23.68	23.70	1.18	1.00	0.86	0.99
Final weight, kg	46.59	47.46	49.21	46.64	49.12	1.92	0.50	0.34	0.83
ADFI, kg/d	1.31 ^ab^	1.31 ^ab^	1.33 ^a^	1.31 ^ab^	1.29 ^b^	0.01	0.04	0.12	0.01
ADG, g/d	360.94	372.27	393.44	358.86	397.13	26.78	0.50	0.34	0.98
F: G	3.67	3.54	3.41	3.64	3.26	0.25	0.46	0.22	0.83

^ab^ Different superscripts in the same row indicate significant differences (*p* < 0.05). ^1^ ADFI = average daily feed intake; ADG = average daily gain; F: G = feed: gain ratio.

**Table 5:** Effects of dietary supplements with tea powder on carcass traits of the pigs.

Item	Tea Powder Inclusion Level					SEM	*p*-Value		
	CON	1%	2%	4%	6%		ANOVA	Linear	Quadratic
Dressing percentage, %	68.25	66.36	68.09	67.45	65.88	1.39	0.35	0.25	0.64
Abdominal fat, kg	1.40 ^a^	1.36 ^a^	1.10 ^b^	1.08 ^b^	1.25 ^ab^	0.12	0.03	0.04	0.04
Carcass length, cm	72.17	75.80	76.40	73.10	74.57	1.65	0.06	0.31	0.14
Skin thickness, mm	2.88	2.61	2.64	2.82	2.80	0.22	0.67	0.92	0.26
Fat thickness, mm	34.56 ^a^	32.01 ^a^	32.98 ^a^	26.14 ^b^	30.53 ^a^	2.01	<0.01	<0.01	0.26
Eye muscle area, cm^2^	9.60	10.37	10.70	9.40	10.27	0.54	0.10	0.78	0.32
Total muscle, %	40.13^b^	39.88^b^	41.70 ^ab^	42.62 ^a^	40.87 ^ab^	0.92	0.03	0.05	0.12
Total fat, %	32.00 ^a^	31.25 ^ab^	27.86 ^c^	26.27^c^	28.45 ^bc^	1.57	<0.01	<0.01	0.07
Total bone, %	12.78	12.72	12.83	13.71	13.31	0.76	0.64	0.22	0.97

^a–c^ Different superscripts in the same row indicate significant differences (*p* < 0.05).

**Table 6:** Effects of dietary supplements with tea powder on meat quality of the pigs.

Item ^1^	Tea Powder Inclusion Level					SEM	*p*-Value		
	CON	1%	2%	4%	6%		ANOVA	Linear	Quadratic
45 min pH	6.29 ^a^	6.25 ^ab^	6.01 ^b^	6.41 ^a^	6.47 ^a^	0.13	0.01	0.06	0.02
24 h pH	5.97	5.91	5.96	5.90	5.92	0.24	1.00	0.84	0.95
Meat color1									
L*	47.29 ^a^	46.68 ^abc^	45.14 ^c^	45.41 ^bc^	46.99 ^ab^	0.83	0.04	0.35	<0.01
a*	14.80	14.97	15.72	15.63	15.36	0.44	0.17	0.08	0.15
b*	5.45 ^ab^	5.59 ^ab^	5.00 ^b^	5.50 ^ab^	5.81 ^a^	0.35	0.04	0.43	0.13
Water-holding capacity, %	80.32	80.17	80.60	80.68	80.75	1.47	0.99	0.67	0.98
Cooking loss, %	43.81	45.39	45.30	45.20	45.35	1.69	0.86	0.44	0.52
Shear force, N	42.47	47.89	45.60	48.64	51.72	4.79	0.41	0.09	0.96
IMF, %	3.39	3.85	3.63	3.83	3.75	0.23	0.26	0.18	0.27
CP, %	21.77	22.42	22.27	22.34	22.42	0.25	0.07	0.04	0.18

^a–c^ Different superscripts in the same row indicate significant differences (*p* < 0.05). ^1^ L*: lightness; a*: redness; b*: yellowness; IMF: intramuscular fat; CP: crude protein.

**Table 7:** Effects of dietary tea powder on free amino acids profile of LD muscle tissue of the pigs.

Item ^1^, (mg/kg)	Tea Powder Inclusion Level					SEM	*p*-Value		
	CON	1%	2%	4%	6%		ANOVA	Linear	Quadratic
Asp	169.52 ^a^	72.50 ^b^	70.22 ^b^	65.16 ^b^	56.43 ^b^	8.35	<0.01	<0.01	<0.01
Thr	40.03 ^b^	51.20 ^a^	43.01 ^b^	44.36 ^ab^	37.02 ^b^	3.66	0.01	0.12	0.01
Ser,	41.50	53.96	45.38	44.64	38.36	5.39	0.07	0.22	0.04
Glu	41.14	54.78	50.90	45.66	46.44	5.16	0.10	0.90	0.05
Gly	182.66	207.77	176.24	174.83	175.55	16.46	0.24	0.22	0.68
Ala	298.49 ^ab^	334.23 ^a^	342.32 ^a^	359.22 ^a^	245.20 ^b^	28.03	<0.01	0.27	<0.01
Val	96.81 ^ab^	100.95 ^a^	102.07 ^a^	88.17 ^ab^	84.55 ^b^	6.42	0.03	0.01	0.08
Cys	9.11	9.88	9.54	8.20	7.23	1.50	0.40	0.11	0.26
Met	15.73 ^c^	23.87 ^a^	22.34 ^ab^	20.09 ^b^	19.73 ^b^	1.66	<0.01	0.26	<0.01
Ile	58.07 ^b^	73.06 ^a^	73.10 ^a^	65.16 ^ab^	63.50 ^ab^	4.49	0.01	0.80	<0.01
Leu	77.06 ^b^	95.52 ^a^	94.64 ^a^	88.46 ^ab^	87.33 ^ab^	6.46	0.05	0.39	0.02
Tyr	52.00	58.61	54.07	57.35	51.16	3.92	0.26	0.75	0.09
Phe	57.28 ^b^	77.88 ^a^	74.66 ^a^	73.68 ^a^	73.70 ^a^	4.24	<0.01	0.01	0.39
Lys	74.04	78.76	77.04	75.66	66.95	4.42	0.09	0.10	0.03
His	37.99	35.79	40.88	38.91	33.99	3.39	0.14	0.35	0.06
Arg	41.01	46.62	42.20	48.03	42.59	3.60	0.24	0.58	0.14
Pro	32.99	38.98	33.23	30.72	28.46	3.90	0.11	0.07	0.11
TAA ^2^	1313.06 ^b^	1428.18 ^a^	1428.74 ^a^	1470.94 ^a^	1328.90 ^b^	31.69	<0.01	0.30	<0.01
EAA ^3^	504.01 ^b^	576.42 ^a^	566.32 ^a^	581.30 ^a^	533.16 ^b^	14.88	<0.01	0.06	<0.01
FAA ^4^	731.94	723.51	755.77	764.71	725.85	21.77	0.23	0.56	0.15
EAA/TAA, %	38.44	40.36	39.67	39.72	40.12	1.31	0.61	0.37	0.49

^ab^ Different superscripts in the same row indicate significant differences (*p* < 0.05). ^1^ Asp: Aspartic acid; Thr: Threonine; Ser: Serine; Glu: Glutamic acid; Gly: Glycine; Ala: Alanine; Val: Valine; Cys: cystine; Met: Methionine; Ile: Isoleucine; Leu: Leucine; Tyr: Tyrosine; Phe: Phenylalanine; Lys: Lysine; His: Histidine; Arg: Arginine; Pro: Proline. ^2^ TAA = total AA. ^3^ EAA = essential AA, including Thr, Val, Met, Ile, Leu, Phe, Lys. ^4^ FAA = flavor AA, including Asp, Glu, Gly, Ala, Met, Arg, Pro.

**Table 8:** Effects of dietary tea powder on fatty acids profile of LD muscle tissue of the pigs.

Item, %	Tea Powder Inclusion Level					SEM	*p*-Value		
	CON	1%	2%	4%	6%		ANOVA	Linear	Quadratic
C10:0	0.10	0.10	0.10	0.09	0.09	0.01	0.33	0.05	0.57
C12:0	0.09	0.09	0.09	0.08	0.08	0.01	0.17	0.02	0.65
C14:0	1.60	1.50	1.55	1.57	1.54	0.07	0.29	0.21	0.78
C15:0	0.05	0.05	0.05	0.05	0.06	0.01	0.67	0.15	0.89
C16:0	27.49	27.58	27.13	27.59	27.37	0.55	0.91	0.85	0.84
C17:0	0.41	0.41	0.42	0.49	0.49	0.05	0.18	0.03	0.58
C18:0	13.08	13.34	13.44	13.66	13.58	0.28	0.27	0.03	0.46
C20:0	0.25	0.23	0.23	0.24	0.25	0.01	0.22	0.70	0.02
C14:1	0.06	0.05	0.05	0.06	0.05	0.01	0.75	1.00	0.64
C16:1	3.73	3.72	3.62	3.70	3.69	0.14	0.94	0.72	0.63
C18:1n9t	0.16	0.15	0.14	0.14	0.14	0.14	0.62	0.26	0.30
C18:1n9c	37.51	38.43	37.82	36.85	36.98	1.09	0.60	0.28	0.50
C20:1	1.26	1.20	1.19	1.22	1.27	0.08	0.79	0.87	0.21
C18:2n6c	8.76 ^b^	8.28 ^b^	10.47 ^a^	9.50 ^ab^	9.85 ^ab^	0.80	0.04	0.07	0.49
C18:3n6	0.08	0.08	0.08	0.08	0.08	0.01	1.00	0.91	0.87
C18:3n3	0.23	0.21	0.25	0.25	0.26	0.02	0.25	0.09	0.44
C20:3n6	0.35	0.34	0.36	0.36	0.37	0.03	0.89	0.34	0.88
C20:2	0.37	0.34	0.36	0.35	0.37	0.03	0.86	0.76	0.49
C20:4n6	3.02	2.83	3.49	3.35	3.30	0.31	0.22	0.13	0.52
C20:5n3	0.09 ^b^	0.09 ^b^	0.09 ^b^	0.13 ^a^	0.17 ^a^	0.02	<0.01	<0.01	0.03
SFA ^1^	43.65	43.36	43.03	43.96	43.46	0.79	0.81	0.90	0.69
MUFA ^2^	42.75	43.54	41.94	41.86	42.13	1.35	0.71	0.33	0.89
PUFA ^3^	13.60	13.10	15.04	14.52	14.42	1.70	0.80	0.42	0.72

^ab^ Different superscripts in the same row indicate significant differences (*p* < 0.05). ^1^ SFA (saturated fatty acid %) = C10:0 + C12:0 + C14:0 + C15:0 + C16:0 + C17:0 + C18:0 + C20:0. ^2^ MUFA (monounsaturated fatty acid %) = C14:1 + C16:1 + C18:1n9t + C18:1n9c + C20:1. ^3^ PUFA (polyunsaturated fatty acid %) = C18:2n6c + C18:3n6 + C18:3n3 + C20:3n6 + C20:2+ C20:4n6 + C20:5n3.

**Table 9:** Effects of dietary supplements with tea powder on serum biochemical indices of the pigs.

Item	Tea Powder Inclusion Level					SEM	*p*-Value		
	CON	1%	2%	4%	6%		ANOVA	Linear	Quadratic
ALT, (U/L)	70.34	62.84	69.52	62.42	68.70	3.64	0.08	0.67	0.16
AST, (U/L)	52.89 ^b^	50.13 ^b^	65.78 ^a^	56.50 ^ab^	56.25 ^ab^	5.44	0.05	0.31	0.17
ALP, (U/L)	124.11 ^b^	129.38 ^ab^	150.60 ^a^	142.25 ^ab^	120.00 ^b^	11.75	0.05	0.87	<0.01
CREA, (μmol/L)	95.10 ^ab^	99.00 ^a^	103.89 ^a^	95.67 ^ab^	84.33 ^b^	6.54	0.05	0.11	0.02
GLU, (mmol/L)	5.13	4.53	5.06	5.11	4.67	0.35	0.29	0.67	0.86
TG, (mmol/L)	0.87	0.83	0.85	0.79	0.85	0.09	0.91	0.69	0.61
CHOL, (mmol/L)	3.09	3.03	2.96	2.99	2.92	0.20	0.92	0.39	0.87
HDL, (mmol/L)	0.97 ^c^	1.14 ^ab^	1.03 ^bc^	1.12 ^abc^	1.25 ^a^	0.07	<0.01	<0.01	0.51
LDL, (mmol/L)	1.89 ^a^	1.70 ^ab^	1.71 ^ab^	1.68 ^ab^	1.49 ^b^	0.13	0.05	<0.01	0.91
LPS, (U/L)	43.52 ^b^	29.28^b^	102.61 ^a^	97.35 ^a^	52.63 ^b^	15.77	<0.01	0.02	<0.01

^a^^–c^ Different superscripts in the same row indicate significant differences (*p* < 0.05).

**Table 10:** Effects of dietary supplements with tea powder on serum antioxidative parameters of the pigs.

Item, (U/mL)	Tea Powder Inclusion Level					SEM	*p*-Value		
	CON	1%	2%	4%	6%		ANOVA	Linear	Quadratic
TAO-C	3.34	3.42	3.24	3.40	3.44	0.09	0.24	0.40	0.29
SOD	90.83	91.91	92.78	90.81	95.68	2.72	0.37	0.16	0.51
GSH-Px	814.17 ^ab^	785.89 ^b^	820.92 ^a^	809.51 ^ab^	831.65 ^a^	13.81	0.03	0.08	0.14
MDA, (nmol/mL)	4.48	4.07	3.79	3.83	3.98	0.33	0.25	0.10	0.11

^ab^ Different superscripts in the same row indicate significant differences (*p* < 0.05).

## Data Availability

Not applicable.

## Abbreviations

ADFI: average daily feed intake; ADG: average daily gain; F:G: the ratio of feed to meat; L*: lightness; a*: redness; b*: yellowness; IMF: the intramuscular fat; CP: crude protein; ALT: alanine transaminase; AST: aspartate transaminase; ALP: alkaline phosphatase; LPS: lipase; CREA: serum concentrations of creatinine; GLU: blood glucose; TG: triglyceride; CHOL: total cholesterol; HDL: high-density lipoprotein; LDL: low-density lipoprotein; TAO-C: total antioxidant capacity; SOD: superoxide diamutase; GSH-Px: glutathione peroxidase; MDA: malondiadehyde; ACC: acetyl-CoA carboxylase α; FATP1: fatty acid transport protein 1; HSL: hormone-sensitive lipase; ATGL: adipose triglyceride lipase.
